# Supplementary material for: Spatiotemporal profiling of modification-specific proteome secretion uncovers an itaconation-activated tyrosine kinase
Source: Nat Commun. 2025 Dec 4;16:10924. doi: 10.1038/s41467-025-66508-y (PMC12686461; doi:10.1038/s41467-025-66508-y)
Supplement: Supplementary file 1 — Description of Additional Supplementary Files [file 41467_2025_66508_MOESM1_ESM.pdf]

Description of Additional Supplementary Files

File Name: Supplementary Data 1.

Description: Chemoproteomic profiling of itaconated secreted proteins identified by PBSP in Raw264.7 cell line. Related to Figure 2 and Figure 3. The ratio for each labeled protein is shown. The ITalk labeling sample was compared to control and proteins with average ratio over 2 and p-value less than 0.05 were assigned as enriched proteins. Complete dataset of all identified and quantified proteins with intensities is shown.

File Name: Supplementary Data 2.

Description: Chemoproteomic profiling of itaconated secreted proteins identified by PBSP in iBMDM cell line. Related to Supplementary Figure 3. The ratio for each labeled protein is shown. The ITalk labeling sample was compared to control and proteins with average ratio over 2 and p-value less than 0.05 were assigned as enriched proteins. Complete dataset of all identified and quantified proteins with intensities is shown.

File Name: Supplementary Data 3.

Description: Chemoproteomic profiling of exosome-dependent itaconatedsecreted proteins identified by PBSP in Raw264.7 cell line. Related to Figure 4. The ratio for each labeled protein is shown. The exosome inhibition and ITalk labeling group(GW4869+ITalk) was compared to ITalk labeling group (ITalk) and proteins with ratio below0.5 and p-value below 0.05 were assigned as exosome-dependent itaconated proteins. Complete dataset of all identified and quantified proteins with intensities is shown.

File Name: Supplementary Data 4.

Description: Chemoproteomic profiling of succinated secreted proteins identified by PBSP in Raw264.7 cell line. Related to Figure 6. The ratio for each labelled protein is shown. The Fumarate-alkyne labeling sample was compared to control and proteins with average ratio over 2 and p-value less than 0.05 were assigned as enriched proteins. Complete dataset of all identified and quantified proteins with intensities is shown.

File Name: Supplementary Data 5.

Description: Chemoproteomic profiling of exosome-dependent succinatedsecreted proteins identified by PBSP in Raw264.7 cell line. Related to Figure 6. The ratio for each labeled protein is shown. The exosome inhibition and Fumarate-alkyne labelling group (GW4869+Fumarate-alkyne) was compared to Fumarate-alkyne labeling group(Fumarate-alkyne) and proteins with ratio below 0.5 and p-value below 0.05 were assigned as exosome-dependent succinated proteins. Complete dataset of all identified and quantified proteins with intensities is shown.

File Name: Supplementary Data 6.

Description: Sequence of primers used for constructing the plasmids.
