# Supplementary material for: Spatiotemporal profiling of modification-specific proteome secretion uncovers an itaconation-activated tyrosine kinase
Source: Nat Commun. 2025 Dec 4;16:10924. doi: 10.1038/s41467-025-66508-y (PMC12686461; doi:10.1038/s41467-025-66508-y)
Supplement: Supplementary file 9 — Supplementary information [file 41467_2025_66508_MOESM9_ESM.pdf]

Supporting Information for

Spatiotemporal profiling of modification-specific proteome secretion uncovers an itaconation-activated tyrosine kinase

Wenjie Lu[1,2,3,4,5#], Yanling Zhang[1,2,3,4,5#], Xinrui Ni[6,7], Pian Wang[6,7], Shentian Zhuang[6,7*], Wei Qin[1,2,3,4,5 *]

[1]The State Key Laboratory of Membrane Biology, Tsinghua University, Beijing, 100084, China. [2]School of Pharmaceutical Sciences, Tsinghua University, Beijing, 100084, China. [3]Tsinghua-Peking Center for Life Sciences, Tsinghua University, Beijing, 100084, China. [4]MOE Key Laboratory of Bioorganic Phosphorus Chemistry & Chemical Biology, Tsinghua University, Beijing, 100084, China. [5]Beijing Frontier Research Center for Biological Structure, Tsinghua University, Beijing, 100084, China. [6]Institute of Translational Medicine, China Pharmaceutical University, Nanjing 211198, China. [7]Center for Infectious Medicine and Vaccine Research, School of Basic Medicine and Clinical Pharmacy, China Pharmaceutical University, Nanjing 211198, China

*Correspondence: weiqin@tsinghua.edu.cn
shentianzhuang@cpu.edu.cn

#These authors contribute equally.

Content

Supporting Figures 3

Supporting Tables.. 9

Supporting Figures

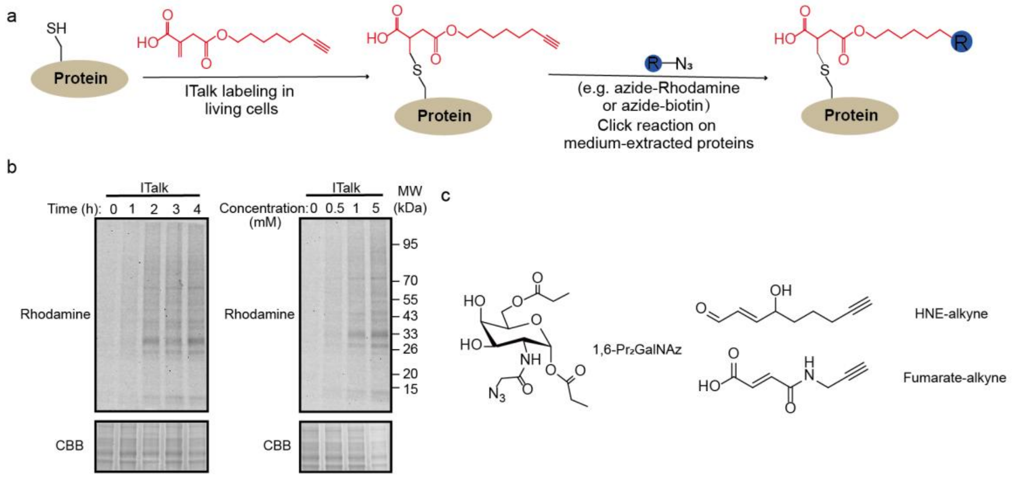

Supplementary Figure 1. Evaluation of ITalk labeling in living Raw264.7 cells. a. Schematic of ITalk labeling targeting the cysteines of itaconated proteins in living cells, followed by click reaction with either azide-Rhodamine or azide-biotin on proteins extracted from the culture medium. b. In-gel fluorescence detection of time-dependent (left) and concentration-dependent (right) ITalk labeling in living Raw264.7 cells. For the optimization of labeling time, Raw264.7 cells were treated with 1 mM ITalk for various durations of time. For the optimization of labeling concentration, Raw264.7 cells were treated with various concentrations of ITalk for 2 hours. The labeled cells were subjected to protein extraction and click reaction with azide-Rhodamine. Labeling intensity was determined by in-gel fluorescence scanning, and Coomassie Brilliant Blue (CBB) staining was used to confirm equal protein loading. Three replicates were performed with similar results. c. Chemical structures of 1,6-Pr_2GalNAz, HNE-alkyne and fumarate-alkyne. Three replicates were performed with similar results. Corresponding uncropped images are shown in the Source Data file.

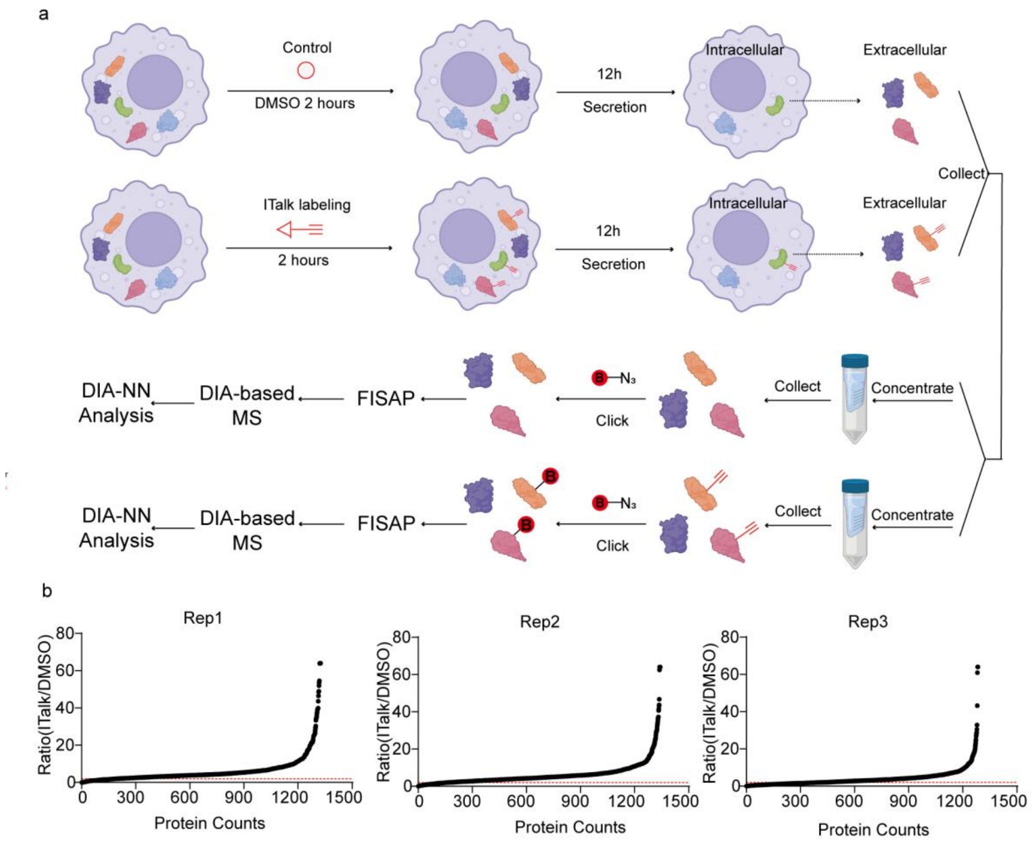

Supplementary Figure 2. Quantitative chemoproteomic profiling of itaconated secreted proteins by PBSP. a. Schematic of PBSP for identifying itaconated secretomes in living macrophages. Macrophages were pulse-labeled with ITalk for 2 hours, followed by probe washout and a 12-hour chase period in serum-free medium. A negative control omitting probe treatment was included. Secreted proteins in the medium were then collected and clicked with azide-biotin. Streptavidin enrichment, on-beads trypsin digestion and desalting were performed by FISAP, followed by DIA-based LC-MS/MS analysis. Created in BioRender. Chu, l. (2025) https://BioRender.com/jj9hibp. b. Distribution of the enrichment ratio of ITalk-labeled secreted proteins in three biological replicates.

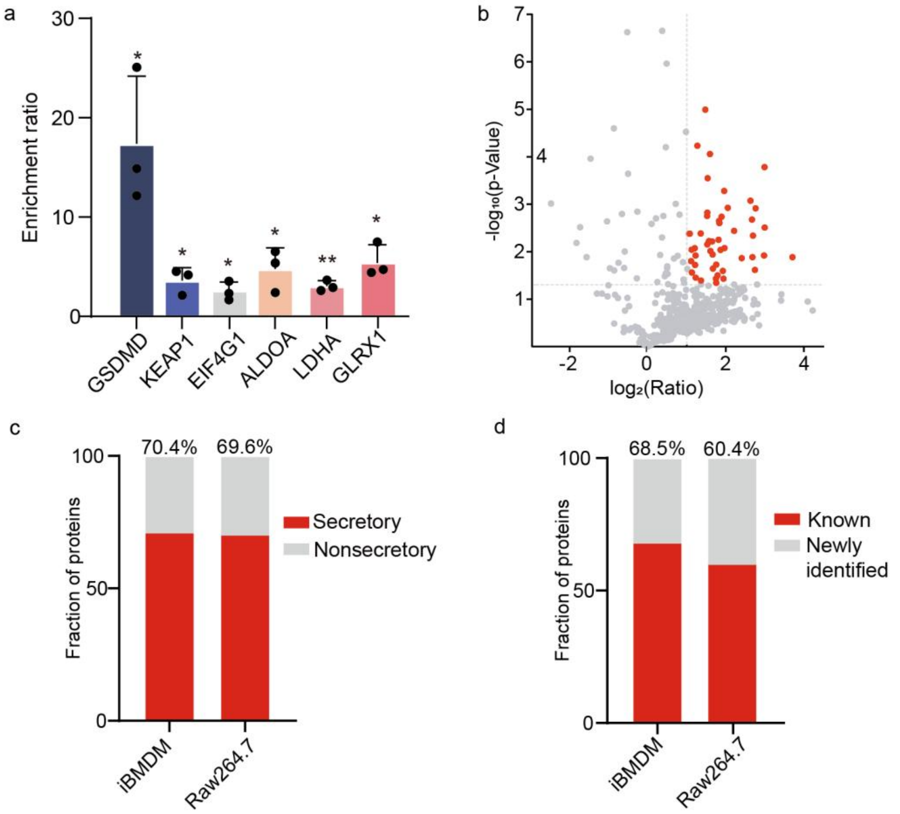

Supplementary Figure 3. Analysis of itaconated secreted proteins in Raw264.7 and iBMDM cell lines. a. Enrichment ratios of representative itaconated secreted proteins in Raw264.7, including GSDMD, KEAP1, EIF4G1, ALDOA, LDHA and GLRX1. *P*-values: 0.01410 (GSDMD), 0.02433 (KEAP1), 0.02598 (EIF4G1), 0.03732 (ALDOA), 0.00285 (LDHA), 0.01739 (GLRX1). The error bars show mean ± SD from three biological replicates. ****p*<0.001 (two-sided student's t-test). b. Volcano plot showing the enrichment of proteins by PBSP in iBMDM cells. Itaconated secreted proteins are highlighted in red. *P*-values were determined by two-sided student t-tests. c. Proportion of known secretory proteins among the itaconated secreted proteins identified by PBSP in Raw264.7 and iBMDM cells. d. Proportion of known itaconated proteins among the itaconated secreted proteins identified by PBSP in Raw264.7 and iBMDM cells.

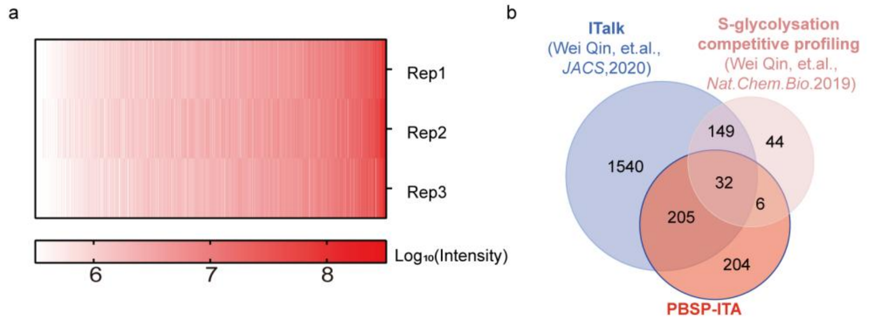

Supplementary Figure 4. Analysis of the exosome-dependent itaconated proteins identified by PBSP. a. Correlation of protein intensities for the exosome-dependent itaconated proteins quantified by the PBSP workflow across three biological replicates. b. Proportion of known itaconated proteins among the exosome-dependent itaconated proteins

identified by PBSP.

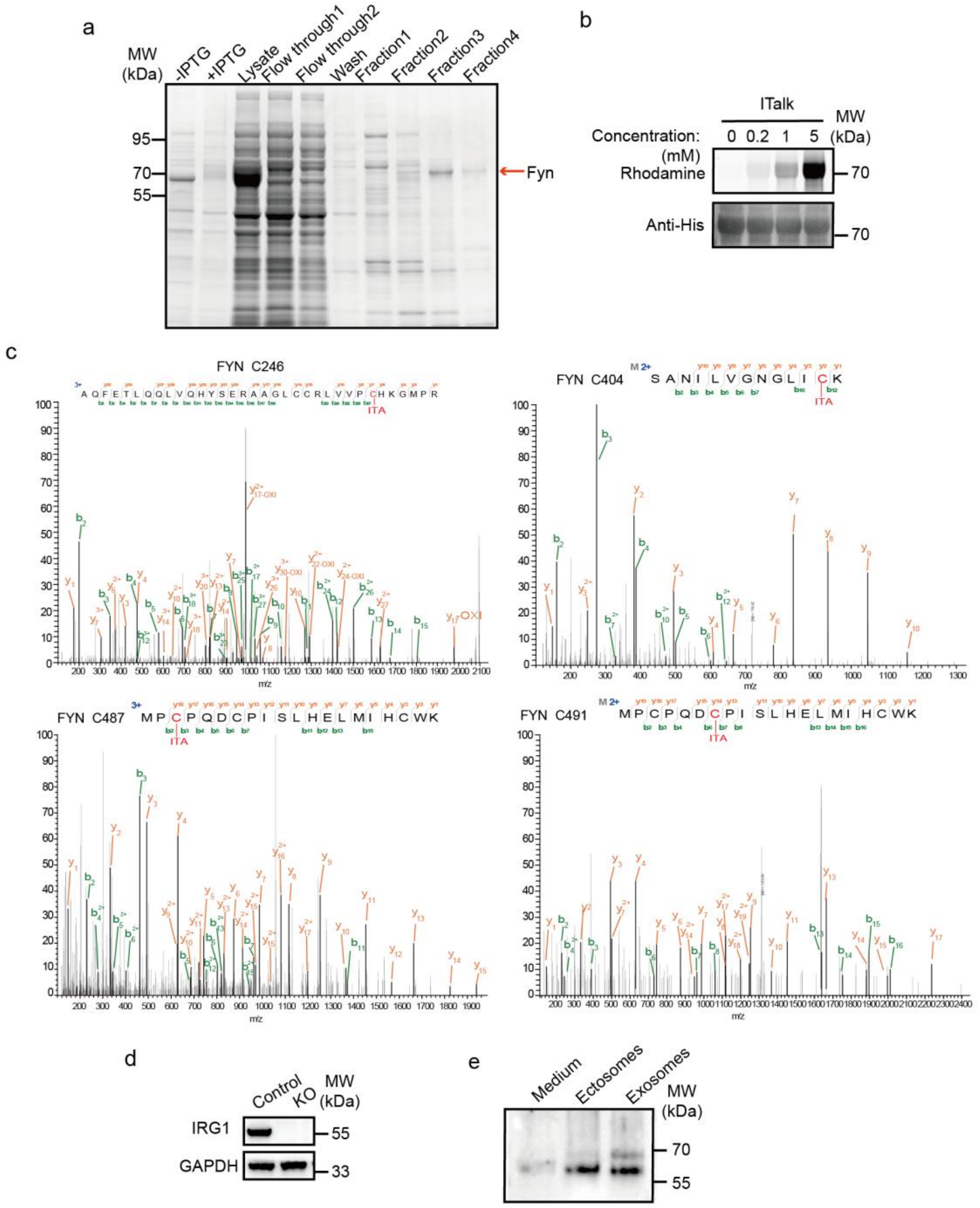

Supplementary Figure 5. Purification and characterization of FYN. a. Purification of His-tagged recombinant FYN from *E. coli*. Fractions 3–4 were concentrated for downstream validation. b. Concentration-dependent FYN itaconation assessed by in-gel fluorescence. Recombinant FYN was incubated with increasing ITalk concentrations (1 hour, 37°C), followed by click chemistry with azide-Rhodamine. Labeling efficiency was quantified by fluorescence scanning; anti-His immunoblotting confirmed equal loading. c. Tandem MS spectra of peptides bearing itaconation on FYN, including the peptide containing Cys246, Cys404, Cys487 and Cys491. d. Validation of *IRG1* knockout in Raw264.7 cells. e. Verification of FYN presence in macrophage-derived extracellular vesicles. Exosomes and ectosomes were isolated from serum-free conditioned medium of Raw264.7 cells (12 hours culture). Three replicates were performed with similar results. Corresponding uncropped images

are shown in the Source Data file.

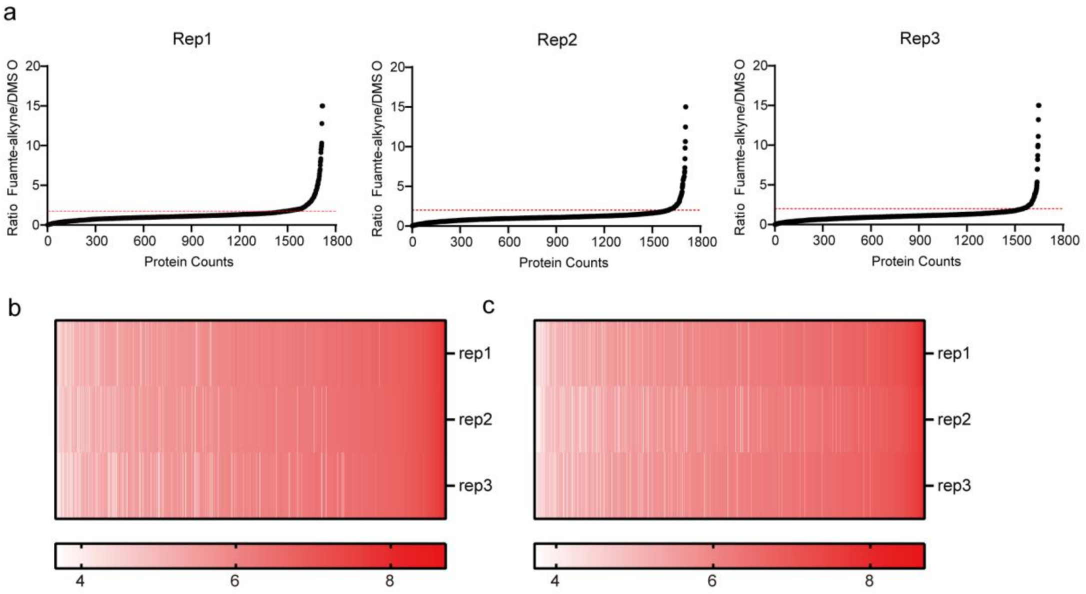

Supplementary Figure 6. Quantitative chemoproteomic profiling of Fumarate-alkyne modified secreted proteins by PBSP. a. Distribution of the enrichment ratio of fumarate-alkyne-labeled secretome proteins in three biological replicates. b. Correlation of succinated protein intensities quantified by the PBSP workflow across three biological replicates. c. Correlation of protein intensities of exosome-dependent succinated proteins quantified by the PBSP workflow across three biological replicates.

Supporting Tables

Supplementary Table 1. Chemoproteomic profiling of itaconated secreted proteins identified by PBSP in Raw264.7 cell line. Related to Figure 2 and Figure 3. The ratio for each labeled protein is shown. The ITalk labeling sample was compared to control and proteins with average ratio over 2 and *p*-value less than 0.05 were assigned as enriched proteins. Complete dataset of all identified and quantified proteins with intensities is shown.

Supplementary Table 2. Chemoproteomic profiling of itaconated secreted proteins identified by PBSP in iBMDM cell line. Related to Supplementary Figure 3. The ratio for each labeled protein is shown. The ITalk labeling sample was compared to control and proteins with average ratio over 2 and *p*-value less than 0.05 were assigned as enriched proteins. Complete dataset of all identified and quantified proteins with intensities is shown.

Supplementary Table 3. Chemoproteomic profiling of exosome-dependent itaconated secreted proteins identified by PBSP in Raw264.7 cell line. Related to Figure 4. The ratio for each labeled protein is shown. The exosome inhibition and ITalk labeling group (GW4869+ITalk) was compared to ITalk labeling group (ITalk) and proteins with ratio below 0.5 and *p*-value below 0.05 were assigned as exosome-dependent itaconated proteins. Complete dataset of all identified and quantified proteins with intensities is shown.

Supplementary Table 4. Chemoproteomic profiling of succinated secreted proteins identified by PBSP in Raw264.7 cell line. Related to Figure 6. The ratio for each labeled protein is shown. The Fumarate-alkyne labeling sample was compared to control and proteins with average ratio over 2 and *p*-value less than 0.05 were assigned as enriched proteins. Complete dataset of all identified and quantified proteins with intensities is shown.

Supplementary Table 5. Chemoproteomic profiling of exosome-dependent succinated secreted proteins identified by PBSP in Raw264.7 cell line. Related to Figure 6. The ratio for each labeled protein is shown. The exosome inhibition and Fumarate-alkyne labeling group (GW4869+Fumarate-alkyne) was compared to Fumarate-alkyne labeling group (Fumarate-alkyne) and proteins with ratio below 0.5 and *p*-value below 0.05 were assigned as exosome-dependent succinated proteins. Complete dataset of all identified and quantified proteins with intensities is shown.

Supplementary Table 6. Sequence of primers used for constructing the plasmids.
